# Characterisation of the Cyanate Inhibited State of Cytochrome c Oxidase

**DOI:** 10.1038/s41598-020-60801-0

**Published:** 2020-03-02

**Authors:** Fabian Kruse, Anh Duc Nguyen, Jovan Dragelj, Ramona Schlesinger, Joachim Heberle, Maria Andrea Mroginski, Inez M. Weidinger

**Affiliations:** 1 Technische Universität Dresden, Department of Chemistry and Food Chemistry, 01069 Dresden, Germany; 2 Technische Universität Berlin, Department of Chemistry, Strasse des 17. Juni 135, 10623 Berlin, Germany; 3 Freie Universität Berlin, Department of Physics, Arnimallee 14, 14195 Berlin, Germany

**Keywords:** Bioenergetics, Biophysical chemistry

## Abstract

Heme-copper oxygen reductases are terminal respiratory enzymes, catalyzing the reduction of dioxygen to water and the translocation of protons across the membrane. Oxygen consumption is inhibited by various substances. Here we tested the relatively unknown inhibition of cytochrome c oxidase (CcO) with isocyanate. In contrast to other more common inhibitors like cyanide, inhibition with cyanate was accompanied with the rise of a metal to ligand charge transfer (MLCT) band around 638 nm. Increasing the cyanate concentration furthermore caused selective reduction of heme *a*. The presence of the CT band allowed for the first time to directly monitor the nature of the ligand via surface-enhanced resonance Raman (SERR) spectroscopy. Analysis of isotope sensitive SERR spectra in comparison with *Density Functional Theory* (DFT) calculations identified not only the cyanate monomer as an inhibiting ligand but suggested also presence of an uretdion ligand formed upon dimerization of two cyanate ions. It is therefore proposed that under high cyanate concentrations the catalytic site of CcO promotes cyanate dimerization. The two excess electrons that are supplied from the uretdion ligand lead to the observed physiologically inverse electron transfer from heme *a*_3_ to heme *a*.

## Introduction

Molecular understanding of the interplay between redox chemistry, oxygen reduction and proton translocation in cytochrome c oxidase (CcO) has been an immense challenge in biophysical science over the last decades. The highly sophisticated coupling of these events comes together at the active site of CcO, containing the heme *a*_3_-Cu_B_ catalytic binuclear center (BNC) and the low spin heme *a* responsible for sequential electron supply. The close vicinity of the two metals (Fe and Cu) within the BNC allows binding of a variety of bridging ligands with different physiological and optical properties. For example, a bridging peroxide (O_2_^2−^) has been proposed to exist in the resting state of CcO^1^ that requires two more electrons to get fully reduced than the oxidized state during catalytic turnover. Intense research has been performed on cyanide^2–7^ (CN^−^) ligands that effectively block the BNC for oxygen. While this inhibition of CcO is a central subject in medical research on cyanide poisoning^8–11^, spectroscopic studies on the binding properties of ligands such as cyanide, azide (N_3_^−^) or sulphide (S^2−^)^2,12,13^ have been performed to derive the oxygen reduction mechanism, to get insight into the structure of the active site^2,5,6,14^ and for spectral isolation of the two heme centers^15–18^. However, in none of these studies so far, the inhibiting ligand in the BNC was directly analyzed via (resonance) Raman spectroscopy.

In the CN-bound state, electron transfer between heme *a* and *a*_3_ is impeded. Preparation of this state has therefore been done to study the structure of central intermediates in CcO^19^. Analysis of the binding kinetics of cyanide to the BNC furthermore led to the discovery of a “slow” and a “fast” form of the enzyme^20–23^. Hereby the “slow” form exhibits lower reactivity than the “fast” form for inhibitors^24^ and, in addition, shows a slowed down intramolecular electron transfer^25^. The “fast” form can be converted reversibly into the “slow” form by lowering the pH value of the buffer solution (to appr. pH6) with concomitant lowering of the protein concentration (<0,4 µM). The corresponding Soret band undergoes a blue shift from approximately 420 nm to 417 nm, which was shown to be caused mainly by the applied pH shift^26^. Interestingly a very similar spectral shift was observed upon formate ligation of CcO in the “fast” form^27^ followed by lowered reactivity towards cyanide. It was thus concluded that both forms differ in their configuration of the BNC. EPR spectroscopy suggested the existence of a bridging ligand in the “slow” form^28^. This suggestion, however, is still controversially discussed^24^.

Cyanate (NCO^−^) originates from oxidation of cyanide but exhibits quite different chemical characteristics. It is considered to be far less toxic than cyanide and has therefore become an important intermediate product in cyanide poisoned water treatment^29,30^. Unique for cyanate is furthermore its ability to dimerize forming the so-called uretdione^31–33^. Cyanate can act as inhibitor for a variety of enzymes^34^ but its interaction with CcO has not been investigated in detail yet. In some early work, inhibition with methyl iso-cyanate was found to lead to a decrease of rat brain CcO activity^35^. This effect was explained by transformation of cyanate to cyanide at that time, but no proof was given based on the structure of the inhibited enzyme. In the present work we have investigated the effects of iso-cyanate inhibition via resonance Raman spectroscopy, which is an exquisite method to derived molecular information on the structure of intermediate states of CcO^36–38^, in combination with quantum chemical calculations^39,40^. Immobilization of CcO on plasmonic Ag electrodes offers the exploitation of surface enhancement^41,42^, which drastically reduces the amount of protein by several orders of magnitude and allows for electrochemical studies^43,44^.

## Methods

The *Rhodobacter sphaeroides* strain JS100 with a C-terminal 6xHis tag at subunit I of the aa_3_-type CcO, was cultivated under aerobic conditions^45^. The enzyme was purified by affinity chromatography as previously described^42,46^. Electrochemically roughened Ag or Au electrodes were incubated in an ethanolic solution of 3 mM 8-aminothiophenol (C8-NH_2_) and 1 mM 6-mercaptohexanol (C6-OH)^47^. For biocompatible immobilization of CcO the coated electrodes were incubated in a solution containing different concentrations of CcO, 10 mM PBS (pH 8.0 and pH6) and 0.1% (w/w) β-DM. The “fast” form of CcO was prepared with concentrations higher than 5 µM in 10 mM PBS buffer at pH 8. The “slow” form of CcO was prepared by lowering the protein concentration to less than 0.4 µM at pH 6 ^21^. Cyanate inhibition was achieved by immersing CcO in buffer solutions containing either KOCN or isotopically labelled KO^13^C^15^N (Cambridge Isotope Laboratories Inc.) of varied concentrations.

The CcO coated Ag or Au ring electrode, immersed in phosphate buffer solution (PBS), was inserted into a homemade electrochemical cell^48^, which was rotated to avoid photoreduction by the laser radiation used for Raman scattering. Surface-enhanced resonance Raman (SERR) experiments were performed with the 413 nm and 647 nm line of a Krypton ion laser (Coherent Innova 300c) or with the 442 nm line of a HeCd laser (VM-TIM, HCL-100). Spectra were recorded using a confocal Raman spectrometer (LabRam HR-800, Jobin Yvon). The laser power on the sample was 150 μW. Accumulation time was 60 s (30 cycles) for each spectrum. UV/Vis spectroscopy of CcO in solution was performed with a Cary 60 spectrometer (Agilent).

Density Functional Theory (DFT) calculations were performed with the BP86 functional^49^ together with the Ahlrichs triple‐zeta polarization all‐electron basis set (TZVP)^50^ for the metals and the 6–31G* basis set for the remaining atoms. Using as starting geometries the coordinates of the BNC extracted from the crystal structure of CcO from *Rhodobacter sphaeroides* (2GSM, chain A), three models were generated by replacing the dioxo ligand bridging the Cu and Fe centers by either cyanate ligands in two different orientations or a bridging uretdione, formed upon dimerization of two cyanate ions. All structural models include the porphyrin ring of heme a_3_ together with the proximal histidine (His-419), the three histidines coordinated to the copper Cu_B_ (His-284/His-333/His-334) and the tyrosine 288. While protein residues were cut at the C_α_–C_β_ bonds, the trimethyltetradecyl tail was cut at the C_11_–C_12_ bond. Atomic vacancies were saturated with hydrogen atoms and in order to avoid large deviations from the initial crystallographic geometry, all C_β_ were frozen to their crystallographic positions. Assuming the enzyme is in the resting state, Fe was modelled in oxidation state 3+ and Cu in oxidation state 2+ conferring the three quantum mechanical models of the BNC a total charge of +2. Geometry optimization and force field calculations were performed with the Gaussian 09 software^51^. Normal mode analysis including computation of vibrational frequencies and corresponding potential energy distribution were performed using software developed in our laboratory^52^. The computation of potential energy distribution (PED) provides quantification and description of the character of a normal mode in terms of the relative contributions of internal coordinates^53^.

## Results

### UV/Vis spectroscopy

**Figure 1:**
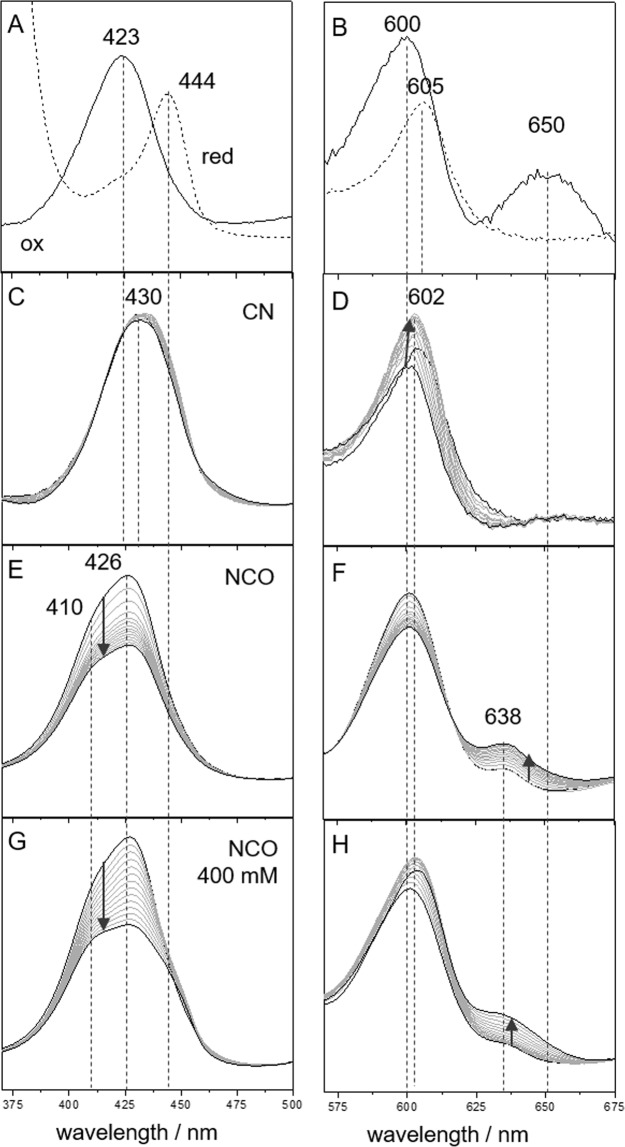
CcO UV/Vis spectra in the Soret and α band in the (**A**,**B**) oxidized (straight) and reduced (dashed) state, after addition of (**C**,**D**) 200 mM CN^−^, (**E**,**F**) 200 mM NCO^−^ and (**G**,**H**) 400 mM NCO^−^. The spectra in (**C–H**) were recorded over a time span of 14 h. The arrows indicate the direction of incubation time.

UV/Vis spectra of CcO (50 µM) were recorded in aqueous solution before and after incubation with KCN or KOCN (Fig. 1). CcO “as prepared” showed typical absorbance maxima at 423 nm (Soret band), 600 nm (α band) and 650 nm (Charge Transfer (CT) complex) indicative of the oxidized state of the protein (Fig. 1A,B)^54^. Incubation with 200 mM KCN (Fig. 1C,D) lead to the well known shift of the Soret band to 430 nm and the disappearance of the CT band at 650 nm^1^. Furthermore a shoulder around 444 nm aroused, which is indicative of the reduced CcO species (Fig. 1A,B dashed line). Spectra recorded every hour over the time span of 14 h showed a slight broadening of both, Soret and α band, together with a slight shift of the latter to 602 nm. Incubation with 200 KOCN (Fig. 1E,F) showed a different behaviour. Here a Soret band shift to 426 nm was observed concomitant with the rise of a shoulder at 410 nm. The CT band at 650 nm disappeared similar to the CN^−^ inhibited enzyme. However, a new band at 638 nm arose. Over the time span of 14 h, the 638 nm and α band gained intensity. Contrary to CN^−^ inhibition no band shifts or band broadening was observed for the α band. At higher KOCN concentrations (400 mM, Fig. 1G,H) the NCO^−^ inhibited state showed additionally a shift of the 600 nm band to 605 nm and the rise of a pronounced shoulder at 444 nm. These band positions are characteristic for the reduced enzyme and suggest at least partial reduction upon NCO^−^ inhibition. The intensity of the 444 nm band in relation to the band at 426 nm increased over time (Fig. S1) reaching saturation after ca. 10 h. Contrary, the band at 638 nm showed continuous increase in intensity and no plateau even after 10 h. Therefore, the data suggests no direct correlation between the presence of the 638 nm band and heme reduction. Furthermore for 400 mM NCO^−^ inhibition a bi-phasic kinetics was observed showing an enhanced increase of the 638 nm band after ca. 6 h (Fig. S1). This observation suggests that a second binding process takes place at this concentration. The 426 nm band remained in the spectrum, which we assign to the inhibited heme a_3_-Cu_B_ site. The 444 nm and 605 nm bands might therefore be indicative of reduced heme *a* only (*vide infra*).

All measurements were done so far with CcO present in its “fast” form, which generally exhibits high binding affinity to inhibitors like CN^−^. If the CcO concentration or the buffer pH is lowered usually the “slow” form of CcO is obtained, in which binding of molecules to the BNC is much less effective^20,21^. Following the preparation procedure from literature, the “slow” form of CcO was generated and subsequently incubated in a NCO^−^ containing buffer solution. Also in this case a shift in the Soret band to 428 nm was observed indicating successful inhibition. However, no band shifts in the UV/Vis spectra characteristic for heme *a* reduction or the rise of the 638 nm band could be detected at any NCO^−^ concentrations (Fig. S2).

### SERR spectroscopy

**Figure 2:**
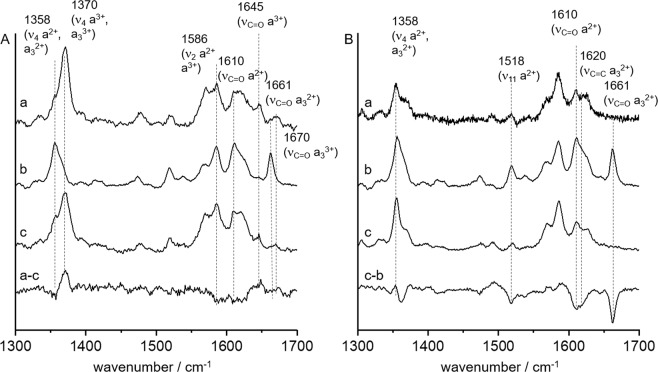
SERR spectra at 413 nm (**A**) and 442 nm (**B**) excitation of immobilized CcO in (a) Phosphate buffer solution (PBS), (b) PBS + dithionite and (c) PBS subsequent to NCO^−^ incubation. The traces a-c and c-b correspond to the respective difference spectra.

To gain insight into the molecular structure of these transitions, the CcO-NCO complex was immobilized on a roughened Ag electrode functionalized with an amino-terminated self-assembled monolayer (SAM)^42,47^ and surface enhanced resonance Raman (SERR) spectroscopy was applied using 413 nm (Fig. 2A) and 442 nm (Fig. 2B) laser excitation, respectively. In trace *a* the corresponding SERR spectra of surface bound CcO in PBS at pH 8 are displayed. Trace *b* shows the spectra of CcO after addition of dithionite to the buffer solution. The spectrum in trace *c* corresponds to the NCO^−^ inhibited protein.

We first consider the (SERR) spectra at 413 nm excitation (Fig. 2A). This excitation line is known to display both oxidation states of the hemes with similar intensity^55^. The SERR spectrum of trace *a* shows strong bands at 1370, 1645 and 1670 cm^−1^ that are characteristic for the oxidized state of heme *a* and *a*_3_, whereas in trace *b* bands at 1358, 1610 and 1661 cm^−1^ dominate. However, contributions from these bands can be already seen in spectrum *a* suggesting that a minor part of the enzyme ensemble is already in its reduced state after surface immobilisation. The spectrum in trace *c* looks like a mixture of *a* and *b*. Indeed, the SERR difference spectrum (a–c) clarifies that the contribution of reduced hemes has increased upon NCO^−^ inhibition. Specifically bands that are indicative of reduced heme a have gained intensity, whereas i.e. the 1661 cm^−1^ band, characteristic for reduced heme a_3_, has not increased. These observations suggest that CNO^−^ binding is accompanied by heme *a* reduction. SERR spectra recorded using 442 nm excitation (Fig. 2B) are only sensitive to the reduced states of the hemes. For the “as prepared” immobilised CcO (trace *a)* only a low intensity spectrum is obtained, which represents the portion of reduced species. Due to the low spectral SERR intensity one might conclude that the majority of the enzyme ensemble is present in its oxidized state. Upon addition of dithionite (trace b) and CNO^−^ inhibition (trace c) the spectral intensity is largely improved indicating that heme reduction has taken place in both cases. Nevertheless the spectra in trace c and b show clear differences most visible in the 1661 cm^−1^ band, characteristic for reduced heme a_3_. As this band is present in trace b but is missing in trace *c*, one can conclude that cyanate inhibition leads to selective reduction of heme *a* only.

**Figure 3:**
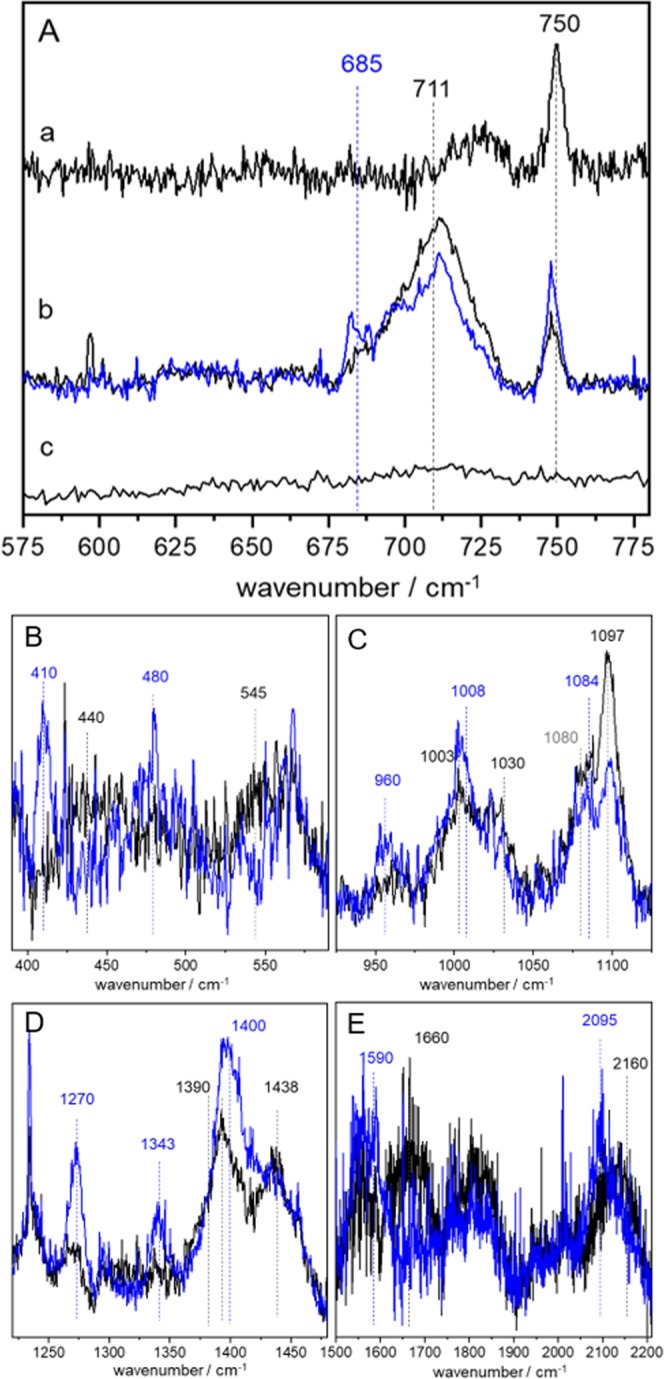
(**A**) SERR spectra recorded with 647 nm excitation before (trace a) and after (trace b) immersion in KOCN (black line) and KO^13^C^15^N (blue line) buffer solution. Trace c shows the “slow” form of CcO in after immersion in KOCN buffer solution. (**B–E**) Same conditions as for A trace b but in an extended frequency range.

To identify the nature of the ligand in the BNC, we performed SERR spectroscopy at 647 nm to selectively excite the metal to ligand charge transfer (MLCT) band of the resting and the NCO^−^ bound state. In Fig. 3A SERR spectra in the wavenumber region between 700 and 900 cm^−1^ before and after NCO^−^ inhibition are shown. For the resting state the corresponding spectrum (trace *a*) features as single band at 750 cm^−1^ previously assigned to the O-O stretching vibration of peroxide^56^. After cyanate addition, this band decreases and a new feature centred at 711 cm^−1^ appears (trace *b*). Interestingly, if the enzyme is in its “slow” form, the 750 cm^−1^ band is vanishing but no new band is appearing (trace *c*). To assign the 711 cm^−1^ band, measurements with isotopically labelled KO^13^C^15^N were performed (trace *b*, blue line). The spectra shown in trace *b* are averaged over several measurements to reduce the signal-to-noise ratio. A reproducible partial shift to 685 cm^−1^ was observed for the isotopically labelled inhibitor (corresponding SERR difference spectra are shown in Fig. S3 in the supporting information). Also in other wavenumber regions isotope shifts were seen (Figs. 3B–E and S3). In the low frequency range (<600 cm^−1^) a weak band at 545 cm^−1^ shifting to 480 cm^−1^ (Fig. 3B) might be rationalised by the data. Furthermore, a shift of a band around 440 cm^−1^ to 410 cm^−1^ can be seen. The region between 900 and 1150 cm^−1^ (Fig. 3C) displayed bands at 1097, 1030 and 1003 cm^−1^ that were shifted to 1084, 1008 and 960 cm^−1^ respectively. In the region between 1200 and 1500 cm^−1^ (Fig. 3D) two isotope sensitive bands at 1390 and 1438 cm^−1^ were seen shifting to 1400 and 1343 cm^−1^ respectively. Furthermore, a band at 1270 cm^−1^ was exclusively seen in the isotopically labelled spectrum. Finally, in the high frequency region (>1500 cm^−1^, Fig. 3E) two broad bands around 2160 and 1660 cm^−1^ were visible that showed a reproducible shift to 2095 and 1590 cm^−1^ upon isotope labelling.

### DFT calculations

**Figure 4:**
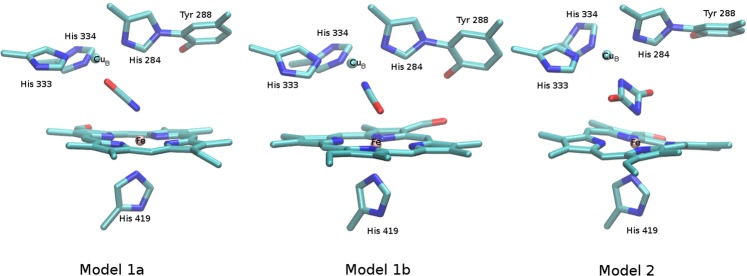
Structural models of the catalytic binuclear center harbouring single cyanate ions (model 1a, model 1b) or an uretdione ligand (model 2). For clarity, hydrogen atoms have been omitted in the representation.

Geometry optimization of the BNC harbouring one cyanate ligand (Fig. 4) bridging the Fe and the Cu metals, leads to a significant increase of the intermetallic distance from 4.89 Å observed in the crystal structure to 5.30 and 5.59 Å in the DFT-optimized models depending on the orientation and binding mode of the cyanate molecule. In particular, a shorter Fe–Cu_B_ distance is predicted when the nitrogen atom of the cyanate is bound to heme *a*_3_ Fe (model 1b). For this model, a lower root mean square deviation (RMSD) of the atomic position computed relative to the crystal structure suggest only minor perturbation of the geometry of the BNC upon NCO^−^ binding (see Table 1, Fig. S4). Interestingly, replacement of the dioxo ligand (present in the crystal structure) by uretdione (Fig. 4, model 2) lead to a lower increase of the Fe…Cu_B_ distance (5.52 Å) as predicted for a single cyanate molecule coordinated to the Fe via its oxygen atom. The reason for this is the compact shape of the uretdione molecule which perfectly fits in the BNC cavity, interacting simultaneously with both metal centers. Interestingly, due to electrostatic repulsion, two separate cyanate ions could not be stabilized within the BNC.

**Table 1:** Selected structural parameters of the three optimized structural models of the BNC harbouring either a cyanate (model 1a, model 1b) ligand or an uretdione ligand (model 2) compared to crystal structure (PDB entry: 2GSM).

	Crystal	Model 1a	Model 1b	Model 2
Ligand	O_2_	(Fe) OCN^−^ (Cu)	(Fe) NCO^−^ (Cu)	(NCO)_2_^−^
RMSD		3.96	0.86	4.59
Fe … Cu_B_	4.89	5.59	5.30	5.52
Fe …OCN^−^ (1a)				
Fe …NCO^−^ (1b)		2.014	1.881	1.852
Fe …NCO_2_^−^ (2)				
Cu …NCO^−^ (1a)				
Cu…OCN^−^ (1b)		1.956	2.184	1.948
Cu …NCO_2_^−^ (2)				
Fe …N(heme a3)	2.065	2.012	2.002	2.026
	2.089	1.992	1.958	2.000
	2.095	2.002	2.036	1.976
	2.135	2.016	2.045	1.978
	(2.096)	(2.006)	(2.010)	(1.995)
Fe …N(H419)	2.109	1.944	1.980	2.026
Cu_B_…N(H284)	2.061	2.041	2.000	2.029
Cu_B_…N(H333)	2.062	2.057	2.051	2.017
Cu_B_…N(H334)	2.080	2.011	2.006	2.088
**Mulliken charges**				
Fe		0.514	0.467	0.532
Cu_B_		0.386	0.408	0.408

Atomic distances as well as root mean square deviation (RMSD) of the position of heavy atoms relative to their crystal arrangement after alignment of Cβ atoms, are given in Å.

In order to elucidate the nature of the ligand, vibrational frequencies and normal modes of vibration were computed for all three models (see Table 2). The PEDs, given in percentage, allowed a precise description of the modes of vibration by estimating the relative contribution of internal coordinates to each normal mode. Thus, only those modes involving internal coordinates, which are potentially affected by a MLCT excitation could be identified and considered for subsequent assignment of experimental vibrational bands.

**Table 2:** Frequencies of vibrational modes (cm^−1^) of the ligand predicted for model 1a, model 1b and model 2 compared to experimental values.

Model 1a Freq.	PED	Model 1b Freq.	PED	Model 2 Freq.	PED	Exp. Freq. [cm^−^¹]
336 (330)	12% Fe-CNO 11% Cu-CNO	362 (361)	14% Fe-CNO 10% Cu-CNO	**485 (465)**	33% Fe- (CNO)_2_ str. 15% Cu- (CNO)_2_ str.	440 (410)
451 (451)	18% Cu-(CNO) def.	424 (422)	25% Fe-CNO			
485 (484)	10% Fe-(CNO) def.	442 (442)	15% Fe-CNO def	**491 (475)**	29% Fe- (CNO)_2_ str.	
**529 (512)**	81% CNO def.	**531 (517)**	40% Cu-CNO def 35% CNO def			545 (480)
**566** **578 (576)**	22% Fe-(CNO) def.	**542 (527)**	59% CNO def 34% Fe-CNO def			
				**669 (639)**	15% (CNO)_2_ def. 12% (CNO)_2_ wagg.	711 (685)
**758**	8% Fe-H419 def.	759	10% Fe-H419 def.	697 700	15% Fe-H419 def.	
				**753 (732)**	40% CN str	
				**962 (926)**	30% CN str 11% (CNO)_2_ sym def	1003 (960) 1030 (1008)
				**1053 (1019)**	15% CN str	
				**1058 (1022)**	23% CN str	
**1058** **1066** **1085**	27% Heme str. 16% Heme str. 13% Heme str.	1050 1070	18% Heme str. 25% Heme str.	1071 1078	20% Heme str. 20% Heme str.	1080
				**1156 (1022)**	6% Cu-N 15% CN str	1097 (1084)
				**1161 (1118)**	35% CN str	
1254 (1233)	57% CO (CNO) str 20% CN (CNO) str	1281 (1259)	52% CN (CNO) str 22% CO (CNO) str			(1270)
				1748 (1705) 1845 (1794)	35% C = O str 46% C = O str	1660 (1590)
**2214 (2135)**	28% CO (CNO) str 72% CN (CNO) str	**2196 (2119)**	32% CN (CNO) str 68% CO (CNO) str			2160 (2095)

PED values reflect the contribution of internal coordination to the corresponding normal mode. In gray, modes involving internal coordination, other than those from the cyanate ligand, which may be enhanced by a MLCT transition. In brackets, vibrational shifts resulting from ^15^N^13^C isotope labelled ligands. Modes that can be assigned to experimental bands are highlighted in bold letters.

## Discussion

### Identification of the ligand

In the case of models 1a and 1b, basically four isotope sensitive vibrational modes associated with the monomer cyanate ligand are predicted (Table 2). Two of them occur between 520 and 580 cm^−1^. We might assign the weak band at 545 cm^−1^ in the experimental spectrum to these vibrations although the intensity is very low and the observed isotope shift is much higher than predicted by theory. Isotope sensitive vibrations at 1254/1281 cm^−1^, as predicted by theory, cannot be found in the experimental spectra with the exception of a band at 1270 cm^−1^ exclusively occurring in the isotopically labelled spectrum. The clearest evidence for the presence of a cyanate monomer is given by the band at 2160 cm^−1^ that coincidences with the predicted vibrations at 2214 or 2196 cm^−1^. Whether the cyanate is bound via the nitrogen (model 1a) or the oxygen (model 1b) cannot be concluded from the data.

On the other hand, strong evidence for the uretdion dimer is given by the isotope sensitive bands at 440, 711, 1003, 1030 and 1097 cm^−1^ that cannot be attributed to the monomer. One might argue that the 440 cm^−1^ band also coincidences with a monomer vibration predicted at 485/442 cm^−1^. However, for the monomer no isotope shift should occur according to the calculations, which contradicts the presence of a clearly shifted band to 410 cm^−1^ in the isotopically labelled spectrum. Therefore this band can be exclusively be assigned to the uretdion dimer. The bands at 711 cm^−1^ and 1097 cm^−1^ overlap with two heme vibrations at 700 and 1080 cm^−1^ that are possibly enhanced by the MLCT, which might explain why we do not see a complete isotope shift of the corresponding bands. In the high frequency region, the C=O vibration is expected to occur at 1748 and 1845 cm^-1^. In the experimental spectrum a very broad isotope sensitive band is seen around 1660 cm^−1^, which might present the two predicted vibrational modes. However, since the position differs quite large and it is not expected that the C=O vibration is enhanced by the MLCT, we defer from a definite assignment.

Finally, there are several isotope sensitive bands visible in the region between 1390 to 1440 cm^−1^ in the experimental spectrum (Fig. 3D) that are not reflected in the simulations. Literature, however, shows in that frequency region a ring stretching vibration for isolated uretdion as well as a stretching vibration for isocyanate^31,57,58^. These vibrations might be the origin for the observed isotope sensitive bands but an assignment to a certain type of ligand is not possible with this data.

In summary, the spectroscopic data together with the DFT simulations strongly indicates that uretdion is at least partially present as a ligand in the BNC. Also evidence of a single cyanate monomer as ligand is given, which leads us to the conclusion that most likely a mixture of these two type of ligands is observed. The presence of two cyanate monomers in the BNC can be discarded since no stable conformation could be found due to the strong repulsive interactions.

### Implications for inverse electron transfer

The cyanate/uretdion inhibited state of CcO features a CT band around 638 nm, which is not observed for the much more common cyanide inhibited state. This feature resembles therefore more the resting state of CcO where a dioxo ligand is present in the BNC and a similar CT band at 650 nm is observed. High concentrations of cyanate show increased reduction of heme *a*. As no extra electrons are available from external donors, reduction of heme *a* can only occur from electrons provided by NCO^−^. As we assume that NCO^−^ can only reach the active site via the oxygen channel, the excess electron has to be delivered via electron transfer from heme *a*_3_ to heme *a*.

Inhibition of the binuclear center (BNC) has been shown in the past using different organic ions such as cyanide (CN^−^), sulfide (S^2−^) or azide (N_3_^−^, which is isoelectronic to NCO^−^)^2–7,12,59^. The involved excess electrons in this process could lead to partial heme reduction and consequently to the rise of the Soret band at around 444 nm^60,61^. In the case of CN^−^, which provides one electron, the band at 444 nm is much weaker than we have observed here with NCO^−^. On the contrary, sulfide, which comes with two excess electrons, leads to a much stronger reduction peak at 444 nm^13^. Based on these observations, we conclude that two excess electrons in the BNC are needed to effectively reduce heme *a*. This can be rationalised as the first electron will most likely reduce rather Cu_B_ than heme *a*_3_ due on its more positive redox potential^36^. The second electron will lead to reduction of heme a_3_ followed by electron transfer (ET) from heme *a*_3_ to heme *a*. Intrinsically heme *a* exhibits a higher redox potential than heme *a*_3_ inhibiting fast ET from heme a to a_3_ prior to proton translocation. Only in combination with oxygen reduction at the BNC and the complex proton pumping process, the redox properties of heme *a*_3_ are changed leading to the physiologically observed ET from heme *a* to *a*_3_. If the electron is, however, delivered directly to heme *a*_3_, proton translocation might not take place in the same way and the initial redox potential difference between heme *a* and *a*_3_ remains. Inverse ET from heme a_3_ to heme a has been observed in CO inhibited CcO subsequent to photolysis of CO^62^. From these experiments it can be concluded that excess electrons from heme *a*_3_ will likely be transferred to heme *a*.

Comparison of the SERR measurements and DFT calculations suggests that in the NCO^−^ bound state either a single NCO^−^ ion or an uretdione can be present as the bridging ligand in the BNC. While both type of ligands will cause a rise of the CT band, only the uretdione is capable of delivering two excess electrons to the BNC and thus can initiate heme *a* reduction. Increasing the NCO^−^ concentration favours uretdione formation, which explains the higher amount of reduced heme *a* when high NCO^−^ concentrations are used. The bi-phasic increase of the 638 nm band in the UV/Vis spectra at high NCO^−^ concentration could indicate that binding of the cyanate monomer is dominant in the beginning of the inhibition process followed by dimerization upon arrival of a second cyanate ion in the BNC.

In the “slow” form of CcO, no formation of a CT band was observed. Nevertheless, the vanishing of the CT band at 650 nm in the UV/Vis spectra together with the decrease of the 750 cm^−1^ band in the SERR spectra suggests that the initial dioxo ligand has been replaced by NCO^−^. Hence, we conclude that NCO^−^ can bind to the BNC in the “slow” state but without the presence of a CT band no SERR spectra, that would give information about the nature of the ligand, can be obtained. However, since the UV/Vis spectra did not show any heme reduction irrespective of the KOCN concentration applied (Fig. S2), we propose that uretdione formation has not taken place. This observation points to different structures and cavities of the BNC in the “fast” and “slow” form.

## Conclusion

Inhibition of CcO with isocyanate lead to the rise of a metal to ligand charge transfer (MLCT) band at 638 nm in the UV/Vis spectrum. A similar MLCT band is visible in the resting state of CcO at 650 nm but has not been observed for other inhibitors yet. Furthermore reduction of heme *a* was observed when high concentration of KCNO was used. The presence of the MLCT band allowed direct monitoring of the ligand via surface enhanced resonance Raman spectroscopy for the first time. The Raman measurements together with DFT calculations suggest that uretdione, formed upon dimerization of two cyanate monomers, is at least partially present as ligand. The dimer delivers two excess electrons to the BNC, where at least one electron is further transferred to heme *a* leading to the observed reduction. Due to the formation of the MLCT band, cyanate inhibition shows great potential for future resonance Raman spectroscopic investigations of the BNC. The additional immobilisation of CcO on electrodes furthermore enables potential dependent surface enhanced Raman spectro-electrochemical investigations that can give insight into redox and protonation events at the active site of CcO.

## Supplementary information

Supplementary information.

## Data Availability

The datasets generated during and/or analysed during the current study are available from the corresponding author on reasonable request.
