# Author Correction: Characterisation of the Cyanate Inhibited State of Cytochrome c Oxidase

**DOI:** 10.1038/s41598-020-63869-w

**Published:** 2020-04-17

**Authors:** Fabian Kruse, Anh Duc Nguyen, Jovan Dragelj, Ramona Schlesinger, Joachim Heberle, Maria Andrea Mroginski, Inez M. Weidinger

**Affiliations:** 1 Technische Universität Dresden, Department of Chemistry and Food Chemistry, 01069 Dresden, Germany; 2 Technische Universität Berlin, Department of Chemistry, Strasse des 17. Juni 135, 10623 Berlin, Germany; 3 Freie Universität Berlin, Department of Physics, Arnimallee 14, 14195 Berlin, Germany

Correction to: *Scientific Reports* 10.1038/s41598-020-60801-0, published online 02 March 2020

The Acknowledgements section in this Article is incomplete.

“We thank Dorothea Heinrich, Jessica Stapel (FU Berlin) and Claudia Schulz (TU Berlin) for the preparation

of CcO samples. We also would like to thank Ernst-Walter Knapp for his support in the calculations and Peter

Hildebrandt for allowing us to measure in his lab. Financial support was provided by the German Research

Foundation through the SFB 1078, projects A1, B4 and C2.”

should read:

“We thank Dorothea Heinrich, Jessica Stapel (FU Berlin) and Claudia Schulz (TU Berlin) for the preparation

of CcO samples. We also would like to thank Ernst-Walter Knapp for his support in the calculations and Peter

Hildebrandt for allowing us to measure in his lab. Financial support was provided by the German Research

Foundation through the SFB 1078, projects A1, B4 and C2. Open Access Funding by the Publication Fund of the

TU Dresden.”

