# Supplementary material for: Characterisation of the Cyanate Inhibited State of Cytochrome c Oxidase
Source: Sci Rep. 2020 Mar 2;10:3863. doi: 10.1038/s41598-020-60801-0 (PMC7052191; doi:10.1038/s41598-020-60801-0)
Supplement: Supplementary file 1 — Supplementary information. [file 41598_2020_60801_MOESM1_ESM.pdf]

SUPPORTING INFORMATION

Characterisation of the Cyanate Inhibited State of Cytochrome c Oxidase

Fabian Kruse[a], Anh Duc Nguyen[b], Jovan Dragelj[b], Ramona Schlesinger[c], Joachim Heberle[c], Maria Andrea Mroginski[b], Inez M. Weidinger[a]*

[a]*Department of Chemistry and Food Chemistry, Technische Universität Dresden, 01069 Dresden, Germany*

[b]*Department of Chemistry, Technische Universität Berlin, Strasse des 17. Juni 135, 10623 Berlin*

[d]*Freie Universität Berlin, Department of Physics, Experimental Molecular Biophysics, Arnimallee 14, 14195 Berlin, Germany*

*author to whom correspondence should be addressed: inez.weidinger@tu-dresden.de

Content

1. UV-Vis Spectroscopy

   Figure S1: Intensity ratios of the 444 nm and 638 nm band vs. the 426 nm band as a function of time.

   Figure S2: UV-Vis spectra of the slow form of CcO as a function of time subsequent to NCO inhibition.

2. SERR Spectroscopy

   Figure S3: SERR difference spectra of CcO incubated with $^{14}N^{12}CO$ minus $^{15}N^{13}CO$ at various frequency ranges using 647 nm excitation.

3. DFT Calculations

   Figure S4: Structural models of the BNC with different cyanate ligands.

1. UV Vis spectroscopy

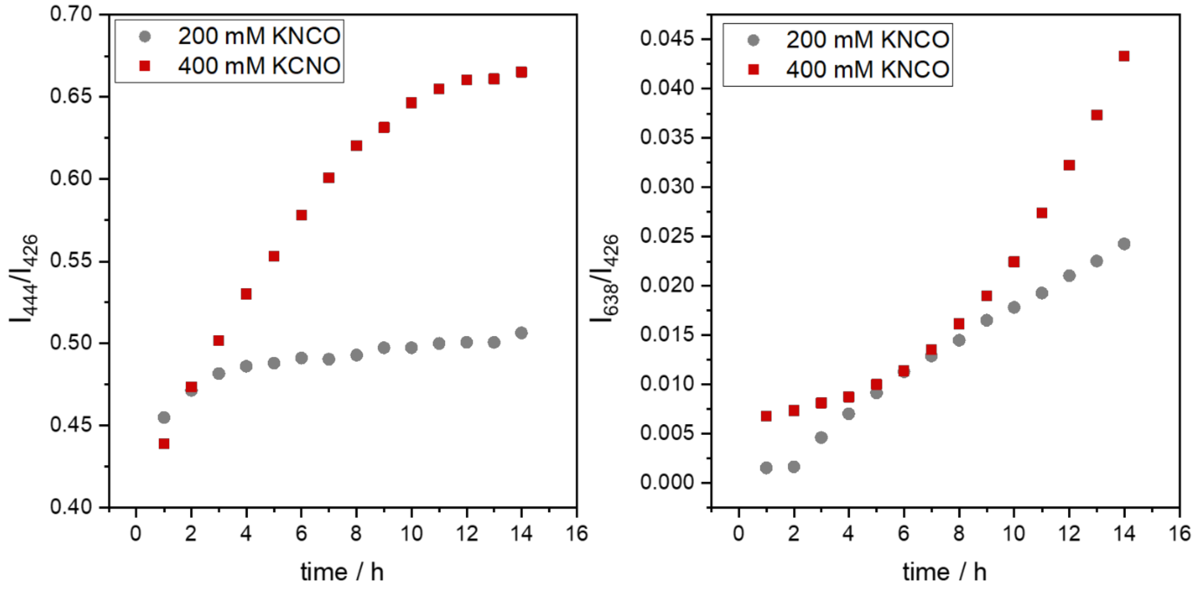

Figure S1: *Intensity ratio of the 444 nm/426 nm (left) and 638 nm/426 nm (right) bands from the spectra in Figure 1 as a function of time.*

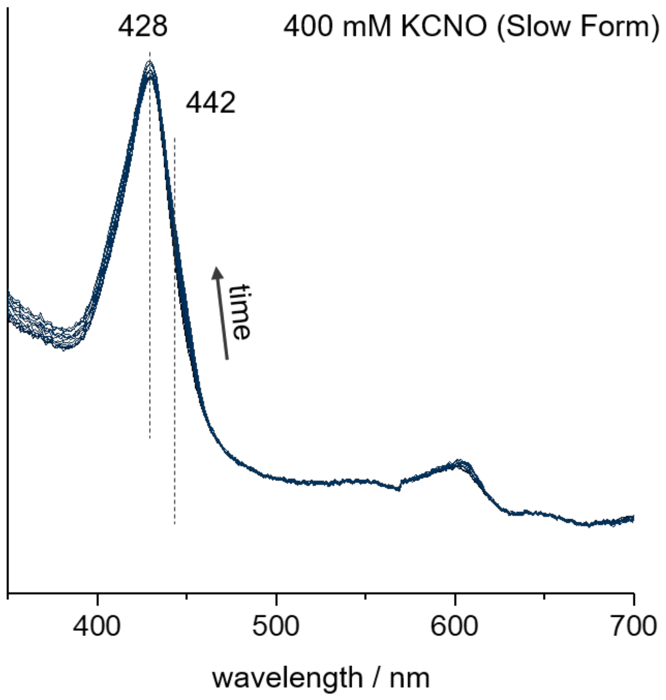

Figure S2: *UV/Vis spectra of the "slow" form of CcO (0.6 μM CcO, 100mM PBS, 0.1% w/w beta-DM) with time (1-14 h) subsequent to incubation with 400 mM cyanate (NCO^-).*

2. SERR Spectroscopy

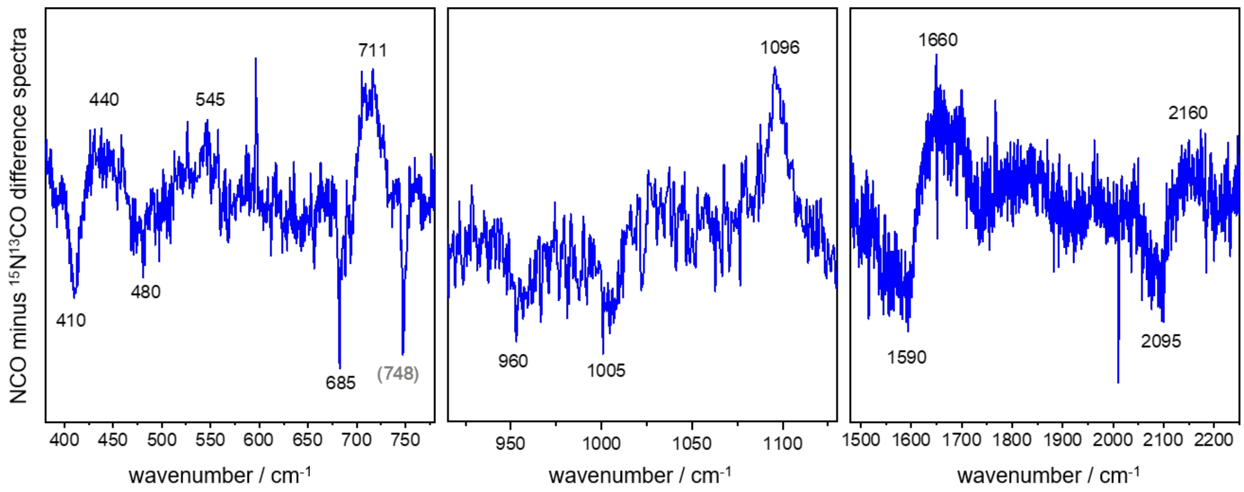

Figure S3: *A: SERR difference spectra of CcO incubated with $^{14}N^{12}CO$ (natural abundance) minus $^{15}N^{13}CO$ (isotopomer) at various frequency ranges. The difference bands can be assigned to vibrations of the cyanate monomer and the uretdion dimer respectively. The difference band at 748 cm^{-1} is characteristic for the resting state and is a result of slightly different cyanate/uretdion binding efficiency for $^{14}N^{12}CO$ and $^{15}N^{13}CO$.*

3. DFT calculations:

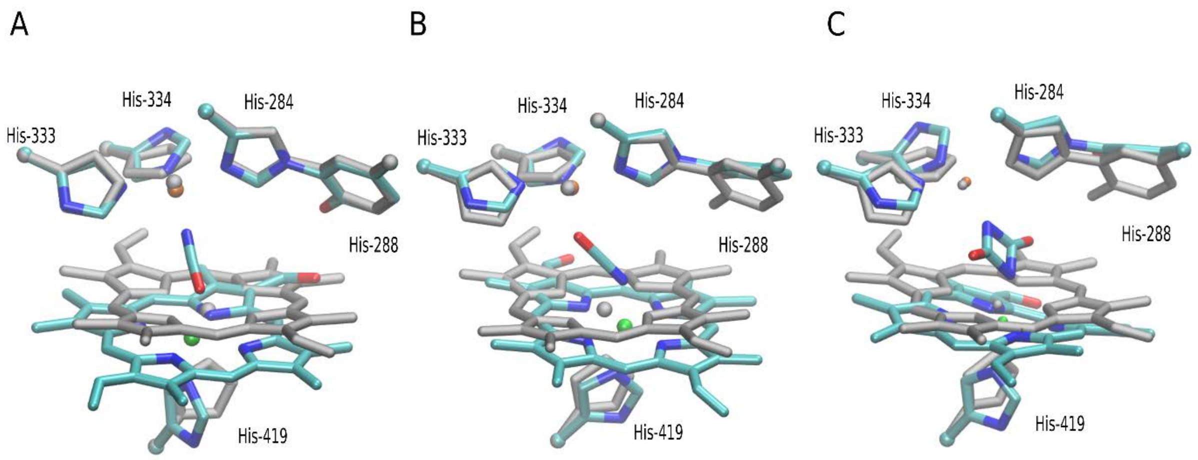

Figure S4: *Structural models of the catalytic binuclear center harboring single cyanate ions (model 1a (A) , model 1b (B)) or an uretdione ligand (model 2, (C)) compared to crystal geometry of CcO extracted from Rhodobacter sphaeroides (2GSM, chain A) depicted in gray. For clarity, hydrogen atoms have been omitted in the representation*
